# Letter from the Editor in Chief

**DOI:** 10.19102/icrm.2023.14056

**Published:** 2023-05-15

**Authors:** Moussa Mansour


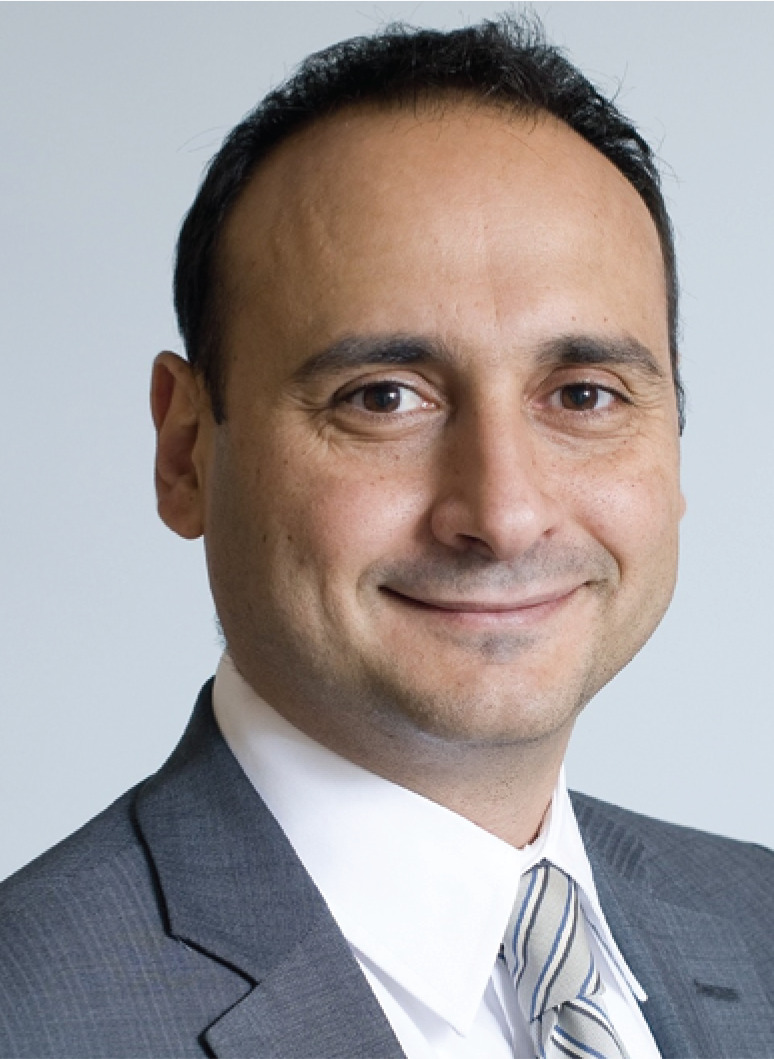


Dear readers,

The annual scientific meeting of the European Heart Rhythm Association was held last month in Barcelona. Important studies were presented at the meeting, and I highlight a few of them in this issue of *The Journal of Innovations in Cardiac Rhythm Management*.

One of the most important presentations given was on the 1-year outcomes of the MANIFEST-PF trial, an observational retrospective study that enrolled post–CE Mark pulsed-field ablation cases performed between March 2021 and May 2022. This study was conducted in 24 European centers and enrolled 1,568 patients treated by 77 operators. The primary effectiveness endpoint was freedom from atrial arrhythmias lasting >30 s during 12 months of follow-up. Sixty-five percent of patients had paroxysmal atrial fibrillation (AF) and the remaining patients had persistent AF. About a quarter of patients underwent additional non–pulmonary vein ablations most commonly involving the posterior wall. During 1 year of follow-up, 10% remained on anti-arrhythmic drugs (AADs) and 78% were free from atrial arrhythmias (including 82% of paroxysmal and 72% of persistent AF cases). This study is the first to report real-world experience with pulsed-field ablation and confirmed the potential of this new energy source.

Another presentation of interest was the Impact of Catheter Ablation Timing on Atrial Arrhythmia Outcomes: Early vs. Delayed Ablation. This was a prospective multicenter trial for ablation of paroxysmal and persistent AF early (within 1 month of randomization) versus after 12 months of treatment with AADs. The primary outcome was freedom from atrial arrhythmias at 12 months of follow-up. Based on prior studies, recurrence rates of 26% in the early ablation group and 55% in the delayed group were predicted, and it was determined that ≥40 patients were required for each study group. Overall, 52 and 48 patients were enrolled in the early and delayed ablation groups, respectively. AF recurrence at 12 months occurred in 43.7% of the early ablation group and 41.4% of the delayed ablation group (*P* = not significant). The main message from this study is that, in centers with long waiting lists for ablation, prescribing an AAD during the waiting period may help to offset the negative effect of waiting long times before undergoing the procedure.

The High RF Power for FASTer and Safer PV Ablation (POWER FAST III) trial was also presented during the conference. During this prospective randomized study, 151 patients were randomized to the high-power, short-duration group (receiving 70 W for 9–10 s) and 144 patients were randomized to the conventional ablation group (receiving 25–40 W guided by ablation index). About two-thirds of patients had paroxysmal AF, and most were treated with AADs prior to ablation. There was no significant difference in procedure time or AF recurrence between the study groups. In a subset study where patients underwent brain magnetic resonance imaging, diffusion-weighted imaging lesions were present in 59% of patients in the high-power ablation group versus 25% of patients in the conventional ablation group (*P* < 0.001). The study findings were surprising, especially when it comes to the magnetic resonance imaging findings and the lack of a difference in procedure duration between the study groups. Larger studies are needed to confirm these findings.

The last study of interest presented was the Adaptive vs. Conventional Cardiac Resynchronization Therapy in Patients with Heart Failure (AdaptResponse) trial. This study analyzed the outcome of the AdaptResponse algorithm in patients with left bundle branch block, aiming to synchronize left ventricular pacing with intrinsic right ventricular conduction in an attempt to reduce unnecessary right ventricular pacing and maximize the use of intrinsic conduction. This was a multinational prospective randomized study that enrolled around 3,617 patients randomized to the new adaptive pacing or conventional cardiac resynchronization therapy pacing. However, the primary endpoint, which was all-cause mortality or heart failure events, was not met, and the study was stopped for futility reasons. Interestingly, a secondary analysis was performed in patients in the adaptive pacing group with >85% and <85% adaptive pacing, respectively, which revealed a 24% reduction in the primary endpoint when >85% adaptive pacing was achieved compared to both conventional cardiac resynchronization therapy and <85% adaptive pacing. It is unclear whether this study will result in significant changes in clinical practice, but it shows at least that adaptive pacing is not inferior to conventional pacing and may result in improved battery life.

I hope that you enjoy reading this issue of *The Journal of Innovations in Cardiac Rhythm Management*.


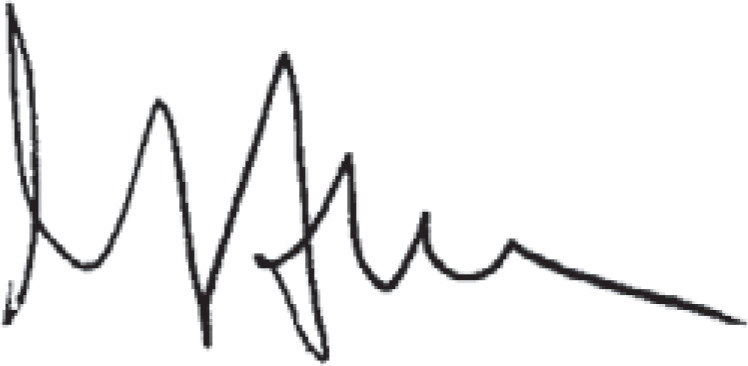


Sincerely,

Moussa Mansour, md, fhrs, facc

Editor in Chief

*The Journal of Innovations in Cardiac Rhythm Management*

MMansour@InnovationsInCRM.com

Director, Atrial Fibrillation Program

Jeremy Ruskin and Dan Starks Endowed Chair in Cardiology

Massachusetts General Hospital

Boston, MA 02114

